# Comparative genomics of *Begonia* chloroplasts: insights into molecular evolution, species identification, and phylogeny

**DOI:** 10.1186/s12864-026-13066-2

**Published:** 2026-07-29

**Authors:** Yang Huang, Wenxiu Tang, Secai Huang, Chao Xiong, Lan Wu

**Affiliations:** 1 College of Tourism and Landscape Architecture, Guilin University of Technology, Guilin, 541006 Guangxi China; 2 Guangxi Zhuang Autonomous Region, Guangxi Institute of Botany, Chinese Academy of Sciences, Guilin, 541006 Guangxi China; 3 School of Life Science and Technology, Wuhan Polytechnic University, Wuhan, 430023 Hubei China; 4 Institute of Chinese Materia Medica, China Academy of Chinese Medical Sciences, Beijing, 100700 China

**Keywords:** *Begonia*, Chloroplast genome, Comparative genomics, Phylogeny, Hypervariable region screening, Identification

## Abstract

**Background:**

*Begonia*, a hyperdiverse genus with ecological and medicinal values, confronts challenges in species identification and phylogenetic resolution due to remarkable morphological plasticity and ambiguous taxonomic boundaries. Chloroplast genomes (plastomes) serve as powerful molecular tools for addressing these issues, yet comprehensive plastomic data for Chinese *Begonia* species remain insufficient. This study presents the largest plastome dataset for Chinese *Begonia* (76 plastomes total), identifies hypervariable markers, and resolves a specific taxonomic issue, clarifying the phylogenetic position.

**Methods:**

We sequenced and assembled plastomes of 25 Chinese *Begonia* species, integrating 51 public plastomes for comparative analyses, including plastome structure, repeat dynamics, codon usage, nucleotide polymorphism, phylogenetics (ML/BI), and positive selection (*Ka/Ks*, BEB tests).

**Results:**

All 25 assembled plastomes exhibited a conserved quadripartite structures (167,365 − 169,901 bp) with 142–143 genes. Eleven hypervariable regions (e.g., *ycf1*, *petB*, *ndhF-rpl32*) were identified as potential DNA barcodes. Phylogenetic tree aligned with geographic distributions, clarifying taxonomic positions (e.g., *B. mashanica*). Five genes (*matK*, *ndhB*, *ndhD*, *rps8*, and *rps15*) showed candidate signals of positive selection, suggesting candidate loci for shade adaptation that require functional validation.

**Conclusion:**

This study enriches *Begonia* plastome resources, provides reliable molecular markers for species authentication, and sheds light on adaptive evolution. The findings support sustainable utilization of medicinal *Begonia* and advancing genus-level evolutionary and taxonomic research.

**Supplementary Information:**

The online version contains supplementary material available at https://doi.org/10.1186/s12864-026-13066-2.

## Introduction

*Begonia* L. (Begoniaceae) is one of the most diverse flowering plant genera, comprising over 2,000 species that thrives across tropical and subtropical regions worldwide [1]. Asia harbors approximately 1,227 species, 35 varieties, and 10 subspecies, with China emerging as a key diversity hotspot [2]. Guangxi ranks second only to Yunnan in species richness, and numerous new taxa have been described from this region recently, including *B. scorpiuroloba* [3], *B. aurora*, *B. larvata* [4], *B. yizhouensis* [5], *B. ufoides* [6] and *B. leipingensis*. This extraordinary diversity, coupled with their adaptation to habitats ranging from karst cliffs to shaded understories, makes *Begonia* a focal group for evolutionary and ecological research.

Beyond their ecological significance, many *Begonia* species have long been valued for medicinal properties and economic potential. In traditional systems, they are used to treat inflammation, cough, hemoptysis, and liver disorders [7]. Modern pharmacological studies have validated these uses, identifying bioactive flavonoids, alkaloids, and phenolics [8]. For example, extracts of *B. multangula* exhibit antimicrobial activity [9], *B. goegoensis* shows antibacterial effects [10], and *B. nantoensis* displays anti-HIV activity [11]. Additionally, *Begonia* species are prized in horticulture, further underscoring the need for accurate species identification and sustainable management [7].

Despite their importance, *Begonia* faces significant taxonomic challenges. Extensive morphological plasticity and overlapping traits (leaf shape, flower structure, and growth habit) often lead to misclassification [12]. This ambiguity complicates authentication of medicinal materials, risking species substitution. Moreover, rapid discovery of new species and unresolved phylogenetic relationships hinder accurate taxonomy [13]. Traditional morphological methods are insufficient, necessitating reliable molecular tools for species authentication and phylogenetic resolution.

Chloroplast genomes (plastomes) have emerged as powerful tools for plant taxonomy and evolutionary studies. Maternally inherited, they exhibit a conserved quadripartite structure (large single-copy [LSC], small single-copy [SSC], and two inverted repeat [IR] regions) with moderate evolutionary rates [14]. Their size ranges (107–218 kb) and gene content (~ 80 proteins, 42 tRNAs, and 8 rRNAs) make them ideal for comparative genomics [15]. Plastomes have been widely applied in species identification, phylogenetics, and detection of adaptive evolution [16, 17]. For *Begonia*, plastome data hold particular promise for resolving fine-scale taxonomic relationships and identifying hypervariable DNA barcodes.

To date, plastome sequencing in *Begonia* has been limited to a small number of representative taxa, such as *B. ferox* [18], *B. handelii* [19], and *B. arachnoidea* [20]. While recent studies have performed comparative analyses within *Begonia*, they either sampled a limited number of species (typically < 10) or did not systematically screen for hypervariable regions across a broad Chinese sample. Consequently, the taxonomic positions of several Chinese *Begonia* species remain ambiguous, and reliable DNA barcodes for species authentication are lacking. To address these gaps, we sequenced and assembled the plastomes of 25 *Begonia* species and combined them with 51 public plastomes for comprehensive analysis. Our specific objectives were to: (a) characterize plastome structur and diversity; (b) identify candidate hypervariable regions as DNA barcodes for species authentication; (c) investigate adaptive evolution in plastome‑encoded genes; and (d) reconstruct a robust phylogeny to resolve taxonomic uncertainties. By integrating these analyses, we aim to provide a molecular framework for accurate species identification, and a deeper understanding of *Begonia* diversification and adaptation.

## Materials and methods

### Plant materials, DNA extraction, and sequencing

Samples of 25 *Begonia* species were collected from the Germplasm Resources Garden of *Begonia* at Guangxi Institute of Botany, China Academy of Sciences, Guangxi Zhuang Autonomous Region (110.17°E, 25.01°N) ( Fig. S1). All plant materials were formally identified by Prof. Wenxiu Tang (Guangxi Institute of Botany, Chinese Academy of Sciences). Voucher specimens for each of the 25 *Begonia* species were deposited in the Herbarium of Guangxi Institute of Botany (IBK), with deposition numbers provided in Table S1. These species were originally sourced from the provinces of Guangxi and Yunnan.

Fresh leaf tissues from each sample were first rinsed with sterile water to eliminate surface impurities, and any moisture was blotted dry using clean absorbent paper. To preserve the integrity of genomic DNA, the treated leaves were immediately desiccated in silica gel. Genomic DNA extraction was carried out by Beijing Gezhi Boya Biotechnology Co., Ltd. DNA concentration, integrity, and purity were initially evaluated using an Agilent 5400 instrument, and further assessments of DNA integrity were conducted with the Agilent 2100 RNA Nano 6000 Assay Kit. After passing all quality control checks, the DNA was randomly fragmented using a Covaris ultrasonic crusher. Library construction was then performed through a series of steps: terminal repair, addition of a 3’-end A-tail, adapter ligation, fragment screening, PCR amplification, and purification of PCR products. The constructed libraries were sequenced on an Illumina Novaseq 6000 platform using 150-bp paired-end (PE) reads, generating a minimum of 4 GB of raw data per species.

### Chloroplast genome assembly

Prior to assembly, raw Illumina paired-end reads were subjected to quality control using fastp v0.23.4 with default parameters: low-quality bases (Phred score < 15) were trimmed from read ends, reads shorter than 50 bp after trimming were discarded, and residual adapter sequences were removed. The resulting clean reads were used for all subsequent analyses. The complete chloroplast genomes of the 25 *Begonia* species were assembled using the GetOrganelle v1.7.8.1 software [21], with *k*-mer value set to 21, 45, 65, 85, 105, 127; all other parameters kept at their default settings. The results of the assembly were visualized using Bandage [22], and successful assembly was confirmed by the presence of a closed circular genome structure. Finally, the assembled sequences were aligned against the chloroplast genome of *B. ferox* (used as a reference) via the online MAFFT tool (https://mafft.cbrc.jp/alignment/server/) to standardize the orientation of each sequence.

### Chloroplast genome annotation

Preliminary annotation of the assembled chloroplast genome was performed using two online tools: CPGAVAS2 [23] and GeSeq [24]. The annotation results were manually refined using Geneious v8.0.2 [25]; this refinement included adjustments of the start and stop codons for each gene and validating annotations by comparing them with the reference genome of *B. ferox*. The orientation of each genome sequence was standardized such that it starts with the large single-copy (LSC) region and ends with the inverted repeat b (IRb) region. The complete chloroplast genome sequences of the 25 *Begonia* species were deposited in the NCBI public database, with accession information provided in Table S1. Visualization of the chloroplast gene structure was achieved using the online tool Organellar Genome DRAW [26].

### Analysis of repetitive sequences and simple sequence repeats

Bioinformatics approaches were employed to systematically analyze repetitive sequences in the chloroplast genomes. Complex repeats were detected using the online REPuter platform [27] with a Hamming distance of 3, this analysis included four types of repeats: forward (F), reverse (R), complementary (C), and palindromic (P) repeats. A minimum sequence similarity of 90% was required for the identification of complex repeats. Simple sequence repeat (SSRs) were identified using the online MISA tool [28] with the following criteria: a minimum of 10 repeats for mononucleotides, 5 repeats for dinucleotides, 4 repeats for trinucleotides, and 3 repeats for tetranucleotides, pentanucleotides, and hexanucleotides. A minimum distance of 100 bp was enforced between adjacent SSRs, and all other parameters were set to default values.

### Comparative analysis of genome boundary regions

The online software CPJSdraw [29] was used to visualize the distribution of gene at the junctions between inverted repeat (IR) regions and single-copy (SC) regions (i.e., LSC and SSC). This visualization enabled the analysis of dynamic changes in IR contraction and expansion across the chloroplast genomes of the 25 *Begonia species*.

### Codon preference analysis

The relative synonymous codon usage (RSCU) of conserved protein-coding sequences in the 25 *Begonia* chloroplast genomes was calculated using CodonW 1.4.2 [30]. An RSCU value greater than 1 indicated a preference for that codon, a value equal to 1 indicated no preference, and a value less than 1 indicated avoidance of the codon. A clustered heatmap illustrating RSCU values across species was generated using TBtools [31].

### Sequence polymorphism analysis

The 76 *Begonia* chloroplast genomes (25 newly sequenced + 51 publicly available) were aligned using MAFFT v7. Nucleotide polymorphism (Pi) was calculated using DnaSP v6.0 [32], with a window length of 600 bp and a step size of 200 bp to assess regional genetic diversity.

### Phylogenetic analysis

To infer phylogenetic relationships among *Begonia* species, two independent datasets were analyzed: the complete chloroplast genomes and the shared protein-coding genes of the 76 species. *Hillebrandia sandwicensis* (Oliv., Begoniaceae) was selected as the outgroup for these analyses. All sequences were standardized to start with the LSC region using Geneious, aligned with MAFFT v7 [33], and trimmed using TrimAl v1.2 [34] with the automated heuristic threshold (“-automated1”) to remove poorly aligned regions. No sites with missing data were removed; gaps were treated as missing data in both ML and BI analyses. For the CDS dataset, missing codons at the ends of alignments were excluded manually. The effective sequence length after trimming was 158,234 bp for the complete plastome dataset and 71,446 bp for the concatenated CDS dataset. Model selection was performed using ModelFinder within PhyloSuite v1.2.3 under the Akaike Information Criterion corrected for small sample sizes (AICc). For the complete plastome dataset, a partitioned strategy was applied: the LSC, SSC, and two IR regions were treated as separate partitions. The best‑fit models were GTR + F+I+G4 for LSC and SSC, and TVM + F+I+G4 for the IR regions. For the concatenated CDS dataset, each of the 78 protein‑coding genes was treated as an independent partition, with the best‑fit model selected individually under AICc. No single model (e.g., TVM + F+I+G4) was applied globally; instead, partition‑specific models were used to avoid model misspecification. Maximum likelihood (ML) phylogenetic trees were constructed with 1,000 bootstrap replicates to assess nodal support [35]. Bayesian inference (BI) trees were built using MrBayes v3.1.2 [36]; posterior probabilities (PP) were sampled every 1,000 generations, and the first 25% of sampled trees were discarded as burn-in to ensure convergence.

### Adaptive evolution analysis

Selection pressures acting on chloroplast protein-coding genes were analyzed across the 76 *Begonia* species. Sequences of 78 conserved protein-coding genes were compared, with Hillebrandia sandwicensis serving as the reference. The ratio of nonsynonymous to synonymous substitution rates (*Ka/Ks*) was calculated using KaKs Calculator v2.0 [37]: a *Ka/Ks* ratio < 1 indicated purifying selection, a ratio = 1 indicated neutral selection, and a ratio > 1 indicated positive selection [38]. if Ks = 0 (no synonymous substitutions), *Ka/Ks* was marked as “NA”. Positive selection sites were further analyzed using EasyCodeML [39], which employs site models [40] and the Bayes empirical Bayes (BEB) method [41]. Amino acid sites with BEB posterior probabilities > 0.95 were considered to be under positive selection. Likelihood ratio tests (LRTs) were performed with the following parameters settings: seqtype = 1, model = 0, NSsites = 0, 1, 2, 3, 7, 8. Amino acid sequences of genes with positive selection signals were visualized using Jalview (v10.0.7), and the structures and domains of these proteins were predicted using the online SWISS-MODEL tool [42], with *B. acetosella* used as an example for visualization. Given that *Ka/Ks* ratios are sensitive to alignment quality and substitution saturation, results with *Ka/Ks* > 1 but restricted to few species pairs were interpreted cautiously, and we prioritized BEB site‑specific tests with posterior probability > 0.95.

## Results

### Plastome composition and structure

**Fig. 1:**
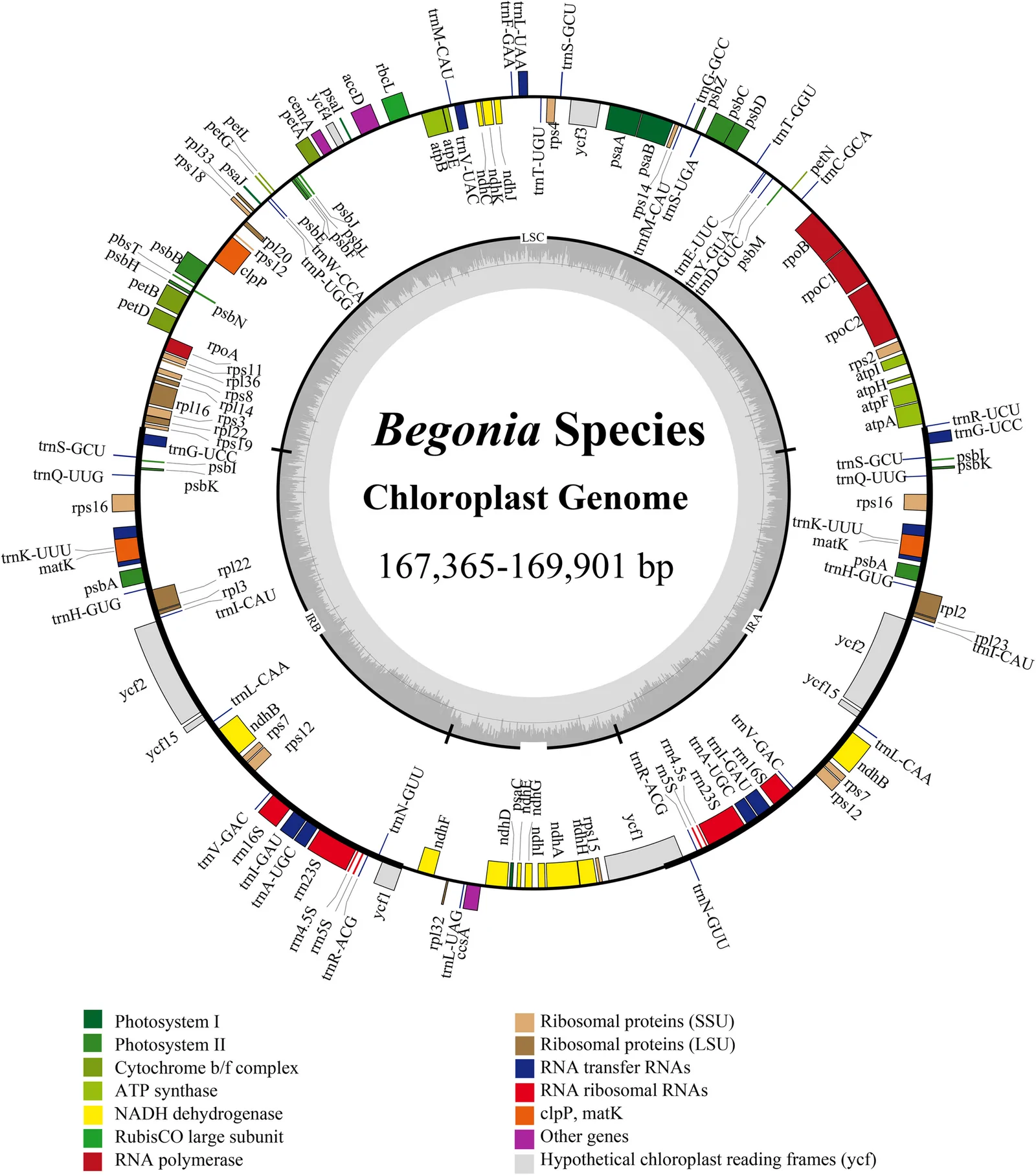
Chloroplast genome structure of *Begonia.* (*B. liuyanii* as an example). The transcription direction of inner ring gene is clockwise, and that of outer ring gene is counterclockwise

All 25 newly sequenced *Begonia* plastomes displayed a typical quadripartite structure, consisting of a large single-copy (LSC) region, a small single-copy (SSC) region, and two inverted repeat (IR) regions. The total length of these plastomes varied from 167,365 bp (*B. acetosella*) to 169,901 bp (*B. curvicarpa*), with the LSC, SSC, and IR regions spanning 73,776 − 76,544 bp, 17,583 − 18,529 bp, and 37,057 − 37,704 bp, respectively (Fig. 1; Table 1). GC content across the plastomes showed slight variation, from 35.42% (*B. pseudodaxinensis*) to 35.89% (*B. larvata*). Notably, IR regions consistently had higher GC content (38.3–38.9%) compared to the LSC (33.6–34.0%) and SSC (27.4–28.8%) regions. Gene annotation revealed that each plastome contained 142–143 genes, including 92–93 protein-coding genes (CDSs), 42 transfer RNA (tRNA) genes, and 8 ribosomal RNA (rRNA) genes. These genes were functionally grouped into four groups: 44 genes related to photosynthesis (e.g., *psaA*,* psbB*, and *ndhF*), 59 genes involved in self-replication (e.g., *rpoA*,* rpl2*, and *trnA-UGC*), 6 genes with other specific functions (e.g., *matK* and *clpP*), and 6 genes of unknown function (e.g., *ycf1* and *ycf2*) (Table 2). Intron analysis identified 15 genes with a single intron (accounting for 10.56% of total genes) and 3 genes with two introns (*clpP*,* ycf3*, and *rps12*; 2.11% of total genes), indicating that splicing mechanisms are conserved within the *Begonia* genus.

**Table 1:** Summary of the chloroplast genomes of 25 *Begonia* species

Species	Total length	LSC length	SSC length	IR length	Total GC content (%)	GC content of LSC (%)	GC content of SSC (%)	GC content of IR (%)	Number of total genes	CDS	tRNA	rRNA
*B. masoniana*	169,108	76,176	18,331	37,364	35.84	33.6	28.33	39	143	93	42	8
*B. retinervia*	169,145	76,064	18,067	37,057	35.53	33.8	28.66	38.9	142	92	42	8
*B. fangii*	169,146	75,925	18,062	37,566	35.49	33.8	28.49	39	142	92	42	8
*B. austroguangxiensis*	169,120	75,881	18,065	37,587	35.49	33.8	28.5	38.9	142	92	42	8
*B. subcoriacea*	168,614	75,901	17,583	37,565	35.76	33.8	28.65	38.9	142	92	42	8
*B. cirrosa*	169,403	76,183	18,106	37,554	35.47	33.8	28.45	38.9	142	92	42	8
*B. liuyanii*	168,498	75,334	18,062	37,551	35.57	34	28.5	38.9	142	92	42	8
*B. luochengensis*	169,473	75,899	18,352	37,611	35.47	33.9	28.37	38.8	142	92	42	8
*B. porteri*	169,075	75,721	18,482	37,436	35.48	33.8	28.29	38.9	142	92	42	8
*B. ningmingensis*	169,104	75,858	18,102	37,572	35.49	33.8	28.47	38.9	142	92	42	8
*B. umbraculifolia*	169,772	76,544	18,088	37,570	35.49	33.8	28.49	38.9	142	92	42	8
*B. curvicarpa*	169,901	76,382	18,091	37,714	35.47	33.8	28.56	38.8	142	92	42	8
*B. wangii*	169,371	75,930	18,283	37,579	35.49	33.9	28.36	38.9	142	92	42	8
*B. filiformis*	169,020	75,856	18,062	37,551	35.5	33.8	28.5	38.9	142	92	42	8
*B. luzhaiensis*	169,031	75,604	18,529	37,449	35.48	33.8	28.2	38.9	142	92	42	8
*B. pseudodaxinensis*	169,528	75,908	18,241	37,704	35.42	32.7	27.43	38.3	143	93	42	8
*B. rhytidophylla*	169,102	75,888	18,080	37,578	35.51	33.7	28.47	38.9	143	93	42	8
*B. linguiensis*	169,177	75,967	18,467	37,372	35.48	33.7	28.16	39	143	93	42	8
*B. larvata*	169,165	75,916	18,062	37,595	35.89	33.7	28.5	38.9	143	93	42	8
*B. edulis*	169,312	75,829	18,283	37,600	35.61	34	28.78	38.9	142	92	42	8
*B. rex*	168,669	75,927	18,306	37,218	35.58	33.9	28.8	38.9	142	92	42	8
*B. truncatiloba*	169,491	76,016	18,279	37,598	35.61	34	28.79	38.9	142	92	42	8
*B. daweishanensis*	169,416	75,934	18,282	37,600	35.61	34	28.78	38.9	142	92	42	8
*B. acetosella*	167,365	73,776	18,323	37,633	35.49	33.7	28.73	38.9	142	92	42	8
*B. mashanica*	169,624	76,437	18,047	37,570	35.5	33.8	28.52	38.9	142	92	42	8

*LSC *large single-copy, *SSC *small single-copy, *IR *inverted repeat, *GC* content, guanine-cytosine content, *CDS *coding sequence, *tRNA *transfer RNA, *rRNA *ribosomal RNA

**Table 2:** Genes of the chloroplast genome of 25 species in *Begonia*

Category	Gene group	Gene name
Photosynthesis	Photosystem I	*psaA*, *psaB*, *psaC*, *psaI*, *psaJ*
	Photosystem II	*psbA#*, *psbB*, *psbC*, *psbD*, *psbE*, *psbF*, *psbH*, *psbI#*, *psbJ*, *psbK#*, *psbL*, *psbM*, *psbN*, *psbT*, *psbZ*
	Cytochrome b/f complex	*petA*, *petB*, petD*, *petG*, *petL*, *petN*
	ATP synthase	*atpA*, *atpB*, *atpE*, atpF*, *atpH*, *atpI*
	NADH dehydrogenase	ndhA*, ndhB*#, *ndhC*, *ndhD*, *ndhE*, *ndhF*, *ndhG*, *ndhH*, *ndhI*, *ndhJ*, *ndhK*
	Rubisco large subunit	*rbcL*
Protein synthesis and DNA- replication	RNA polymerase	*rpoA*, *rpoB*, *rpoC*1*, *rpoC*2
	Large ribosomal proteins	*rpl*14, *rpl*16*, *rpl*2*#, *rpl*20, *rpl*22, *rpl*23#, *rpl*32, *rpl*33, *rpl*36
	Small ribosomal proteins	*rps*11, *rps*12**#, *rps*14, *rps*15, *rps*16*#, *rps*18, *rps*19, *rps*2, *rps*3, *rps*4, *rps*7#, *rps*8
	Transfer RNAs	trnA-UGC*#, *trnC-GCA*, *trnD-GUC*, *trnE-UUC*, *trnF-GAA*, *trnG-GCC*, trnG-UCC*#, *trnH-GUG#*, *trnI-CAU#*, trnI-GAU*#, trnK-UUU*#, *trnL-CAA*#, trnL-UAA*, *trnL-UAG*, *trnM-CAU*, *trnN-GUU#*, *trnP-UGG*, *trnQ-UUG#*, *trnR-ACG#*, *trnR-UCU*, trnS-GGA, *trnS-GCU*#, *trnS-UGA*, *trnT-GGU*, *trnT-UGU*, *trnV-GAC#*, trnV-UAC*, *trnW-CCA*, *trnY-GUA*, *trnfM-CAU*
	Ribosomal RNAs	*rrn*16*S*#, *rrn*23S#, *rrn*4.5*S*#, *rrn*5*S*#
Other genes	Maturase	*matK*#
	Protease	clpP**
	Envelope membrane protein	*cemA*
	Acetyl-CoA carboxylase	*accD*
	c-type cytochrome synthesis gene	*ccsA*
	Translation initiation factor	*infA*[1,2,5]
Genes of unknown function	Conserved hypothetical chloroplast ORF	*ycf*1*#*, *ycf*15*#, *ycf*2*#*, *ycf*3**,* ycf*4

Note: * indicates genes with only one intron; ** indicates genes with two introns; # indicates genes with multiple copies; [] indicates genes present only in certain species. Species 1 *B. larvata;*2 *B. pseudodaxinensis*; 3 *B. masoniana;* 4 *B. rhytidophylla;* 5 *B. linguiensis*

### Analysis of repeat sequences

**Fig. 2:**
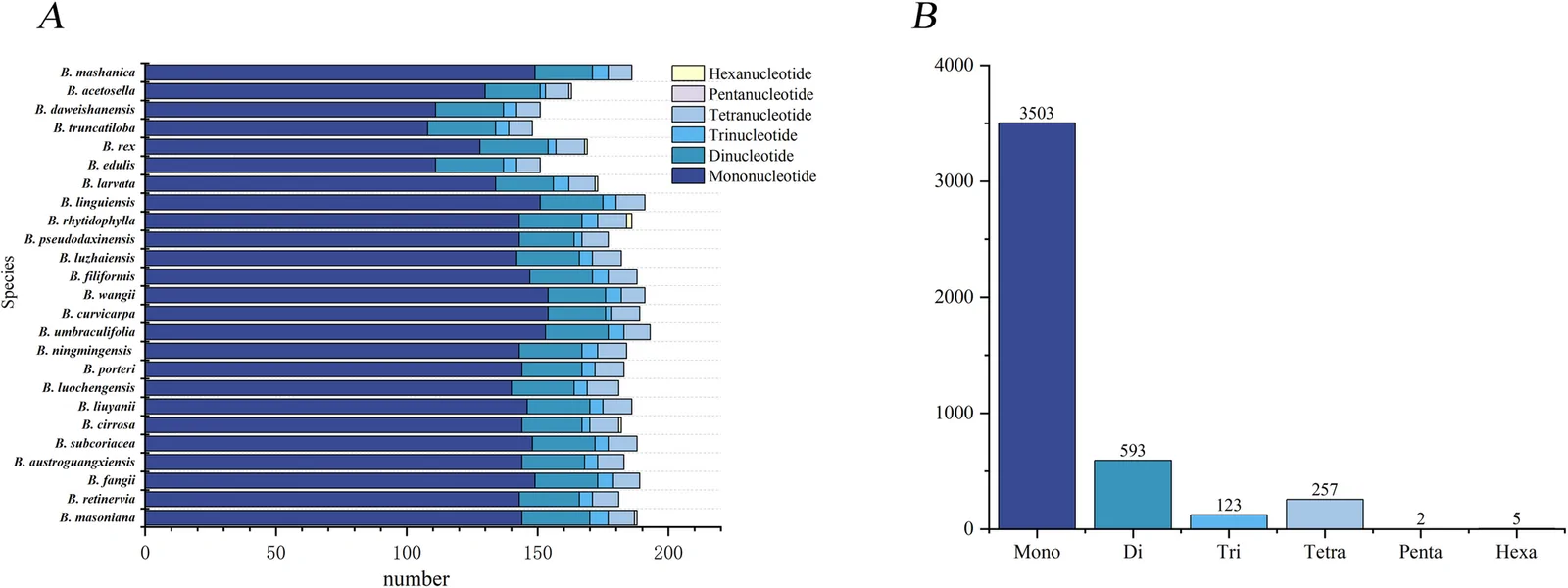
SSR repeat types of 25 *Begonia* species. A: SSR repeat number of 25 species of *Begonia*; B: Number of SSR repeat types

Using the MISA tool, a total of 4,483 simple sequence repeats (SSRs) were detected across the 25 *Begonia* plastomes. The number of SSRs per species ranged from 148 (*B. truncatiloba*) to 193 (*B. umbraculifolia*) (Fig. 2A). Mononucleotide repeats were the most abundant (3,503 SSRs, 78.14%), followed by dinucleotide repeats (593, 13.23%), tetranucleotide repeats (257, 5.73%), trinucleotide repeats (123, 2.74%), hexanucleotide repeats (5, 0.11%), and pentanucleotide repeats (2, 0.04%) (Fig. 2B). Notably, pentanucleotide repeats were exclusively detected in *B. masoniana* (AATAT/ATATT) and *B. acetosella* (AAAAT/ATTTT), indicating species-specific SSR dynamics. It should be noted that these repeat analyses are descriptive, and no statistical comparisons among species or clades were conducted.

Nucleotide composition analysis revealed a strong bias toward A/T-rich motifs, which accounting for 96.92% of all SSRs (Table S2 and S3). For instance, 3,483 out of 3,503 mononucleotide repeats were A/T-based; dinucleotide repeats were dominated by AT/TA motif (518, 11.55%); and tetranucleotide repeats were primarily composed of AAAT/ATTT (215, 4.80%).

**Fig. 3:**
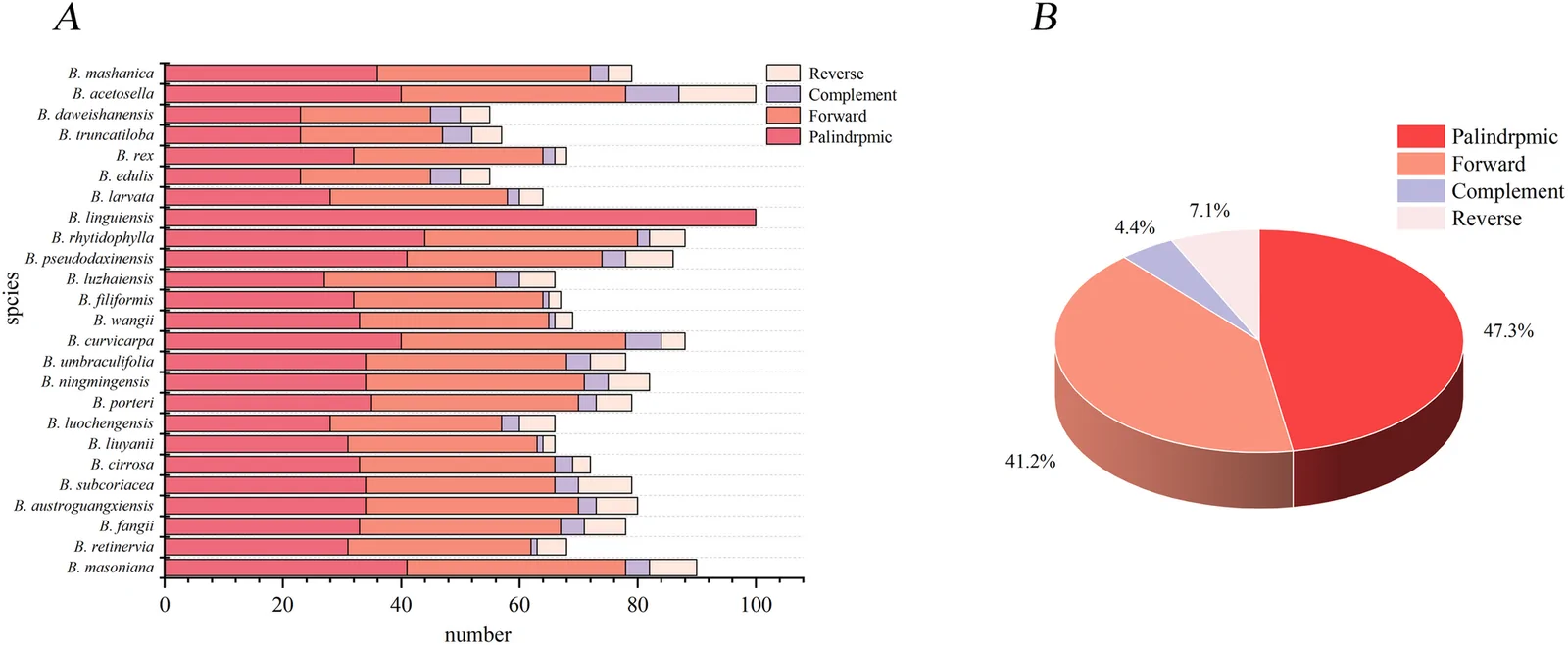
Complex repeat sequence of 25 *Begonia* species. A: Comparison of complex repeat types of 25 species of *Begonia*; B: proportion of complex repeating types

Using the REPuter platform to analyze complex repeats, 55–100 repeats were identified per plastome (Fig. 3A, Table S4). Palindromic repeats (P) were the most common (890, 47.34%), followed by forward repeats (F; 774, 41.17%), reverse repeats (R; 133, 7.07%), and complementary repeats (C; 83, 4.41%) (Fig. 3B). A unique pattern was observed in *B. pseudodaxinensis*, which only contained palindromic repeats—whereas all other 24 species harbored all four repeat types.

### IR/SC boundary analysis

**Fig. 4:**
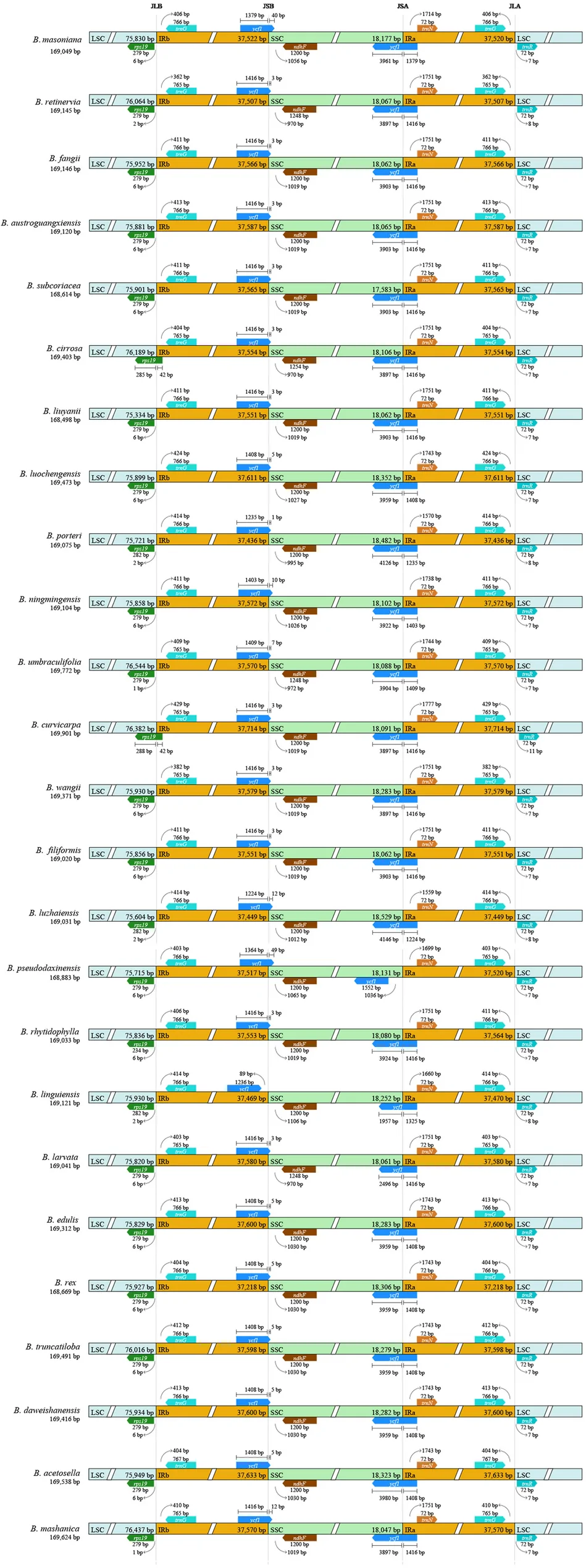
Comparative analysis of plastome boundaries in 25 *Begonia* Species

The boundaries between IR and SC regions (i.e., IRb-LSC [JLB], IRb-SSC [JSB], IRa-SSC [JSA], and IRa-LSC [JLA]) were highly conserved across the 25 *Begonia* plastomes, with only minor lineage-specific variations (Fig. 4). At the JLB boundary (flanked by *rps19* and *trnG*), the junction lay between these two genes in 23 species. However, in *B. cirrosa* and *B. curvicarpa*, the JLB boundary was located within the *rps19* gene. At the JSB boundary (adjacent to *ycf1* and *ndhF*), the junction resided within the *ycf1* in 24 species. In *B. linguiensis*, however, a truncated *ycf1* gene was positioned 89 bp away from the JSB boundary. At the JSA boundary, 24 species showed the junction within *ycf1*, while *B. pseudodaxinensis* had its *ycf1* gene 1,036 bp distal to the JSA boundary. The JLA boundary was strictly conserved across all species, located between t*rnG* (362–429 bp upstream) and t*rnR* (7–8 bp downstream).

### Codon usage bias analysis

**Fig. 5:**
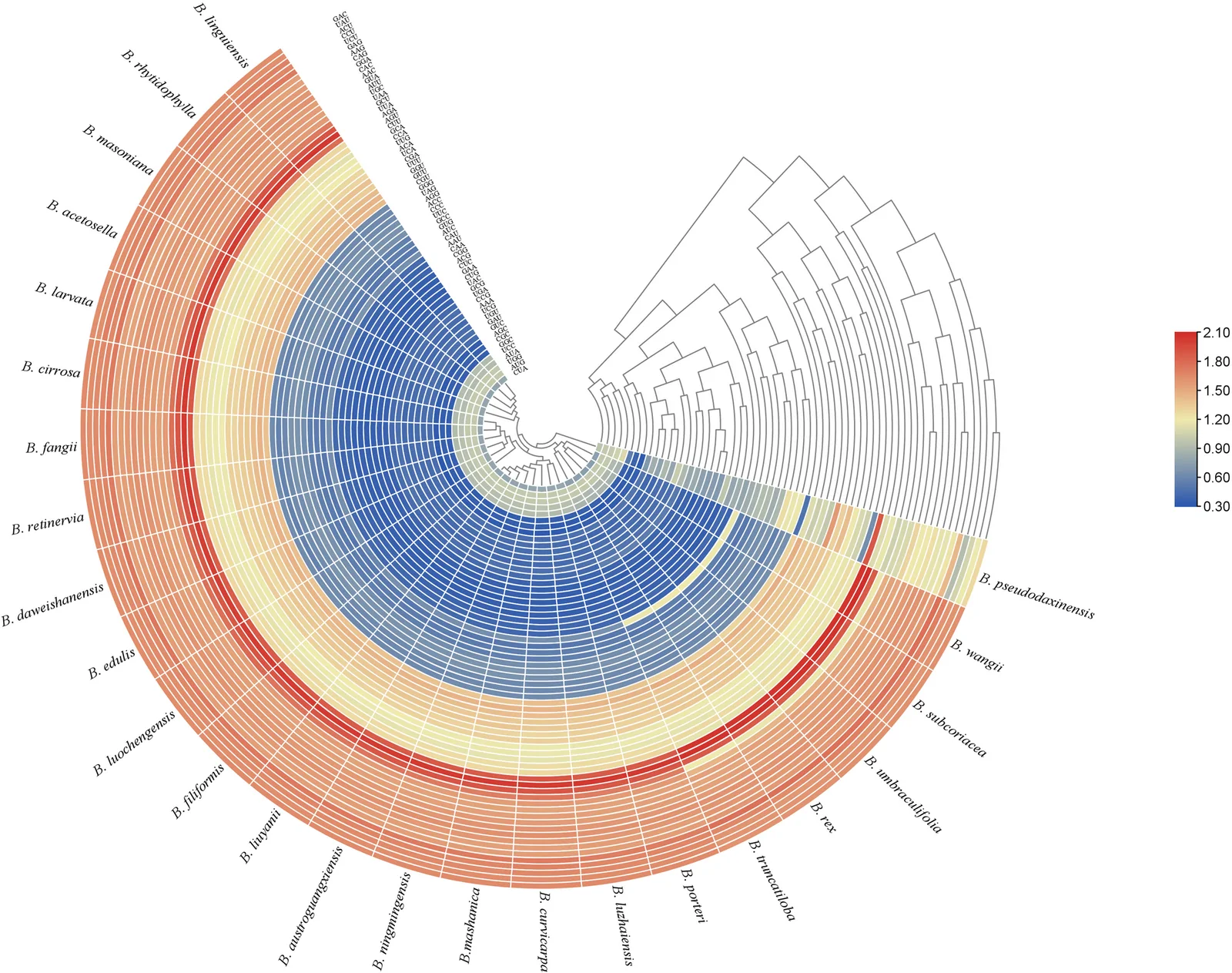
Relative synonymous codon usage (RSCU) of 64 codons across 25 *Begonia* plastomes. Codons are grouped by amino acid (rows); each column represents one species. RSCU > 1 (preferred codons) are shown in red, RSCU < 1 (avoided codons) in blue. The strong bias towards A/T-ending codons is evident across all species

Analysis of 78 conserved protein-coding genes across the 25 plastomes identified 64 synonymous codons encoding 20 amino acids, with total codon counts per genome ranging from 22,015 to 22,393. Relative synonymous codon usage (RSCU) analysis revealed strong and consistent codon preference patterns across all species **(**Fig. 5**)**. Across all species, codons ending with A or U (NNU/NNA) were preferentially used (RSCU > 1), whereas codons ending with G or C (NNG/NNC) were consistently avoided (RSCU < 1). For example, leucine was encoded preferentially by UUA (RSCU = 1.86 ± 0.05) and UUG (1.21 ± 0.04), while CUC (0.53 ± 0.03) and CUG (0.38 ± 0.04) were strongly avoided. Similarly, isoleucine showed strong preference for AUU (1.58 ± 0.04) over AUC (0.70 ± 0.03). This A/U-ending bias was observed for all 20 amino acids. The codon usage profile of *B. pseudodaxinensis* showed consistently lower RSCU values across most codons compared to congeners (average RSCU difference = 0.12 ± 0.03, paired t-test *p* < 0.01). Additionally, the termination codons UGA and UAA were less frequently used in *B. wangii*, *B. subcoriacea*, *B. umbraculifolia*, *B. rex*, and *B. truncatiloba*, indicating lineage-specific variation in translation termination efficiency.

### Sequence polymorphism analysis

**Fig. 6:**
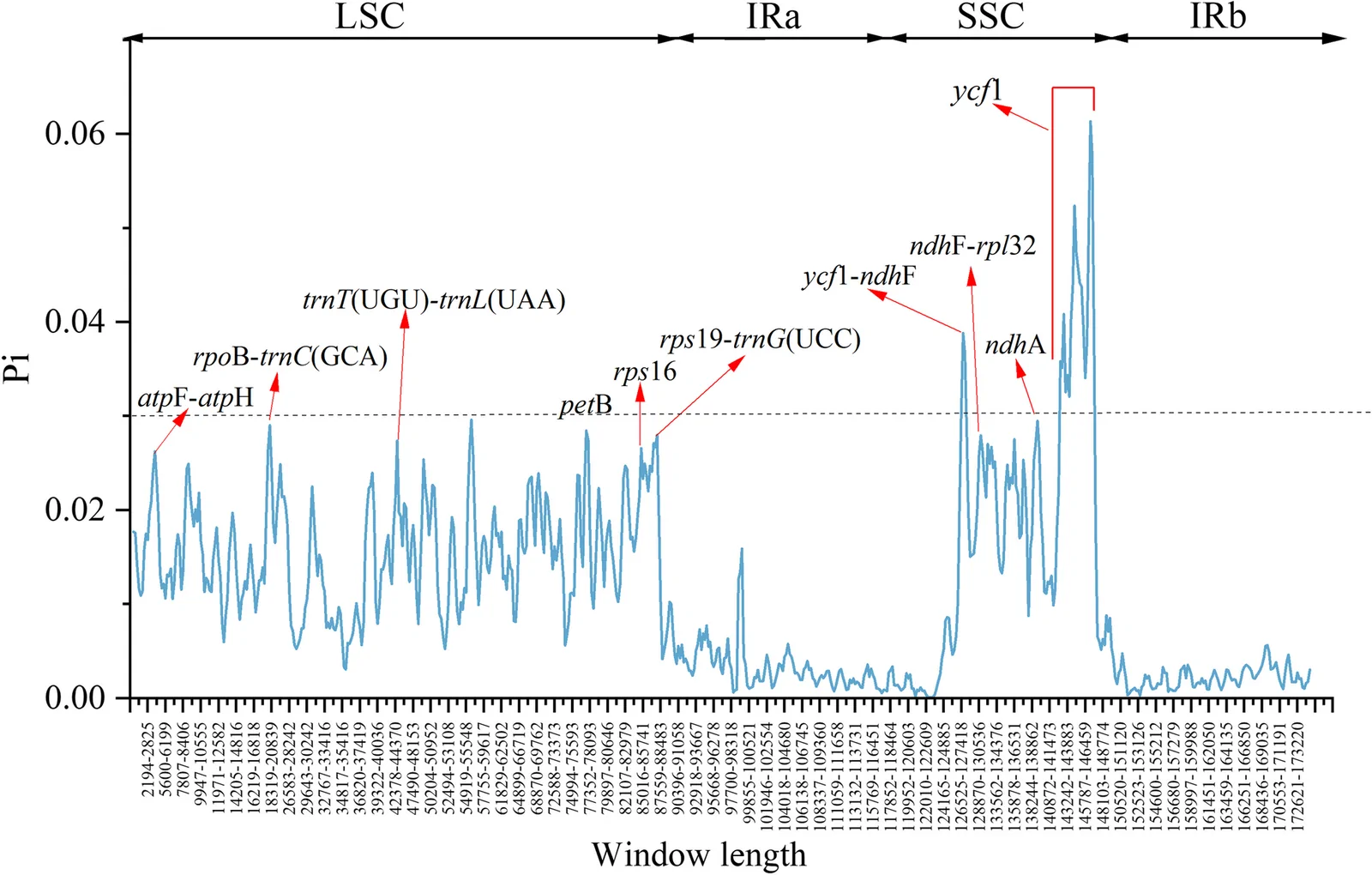
Nucleotide variability (Pi) of chloroplast genome sequences of 25 *Begonia* plants

Nucleotide polymorphism (Pi) analysis of 76 *Begonia* plastomes (including 25 newly sequenced and 51 publically available) revealed significant regional difference in genetic diversity (Fig. 6). The SSC region exhibited the highest average Pi value (0.02634), followed by the LSC region (0.01555), while the IR regions were highly conserved (average Pi = 0.00355). Four protein-coding genes showed high polymorphism: *petB*, *rps16*, *ndhA*, and *ycf1*. Additionally, seven intergenic spacers displayed elevated Pi values: *atpF-atpH*, *rpoB-trnC-GCA*, *trnT-UGU-trnL-UAA*, *rbcL-accD*, *rps19-trnG-UCC*, *ycf1-ndhF*, and *ndhF-rpl32* (the latter two are localized in the SSC region).

### Phylogenetic relationships analysis

**Fig. 7:**
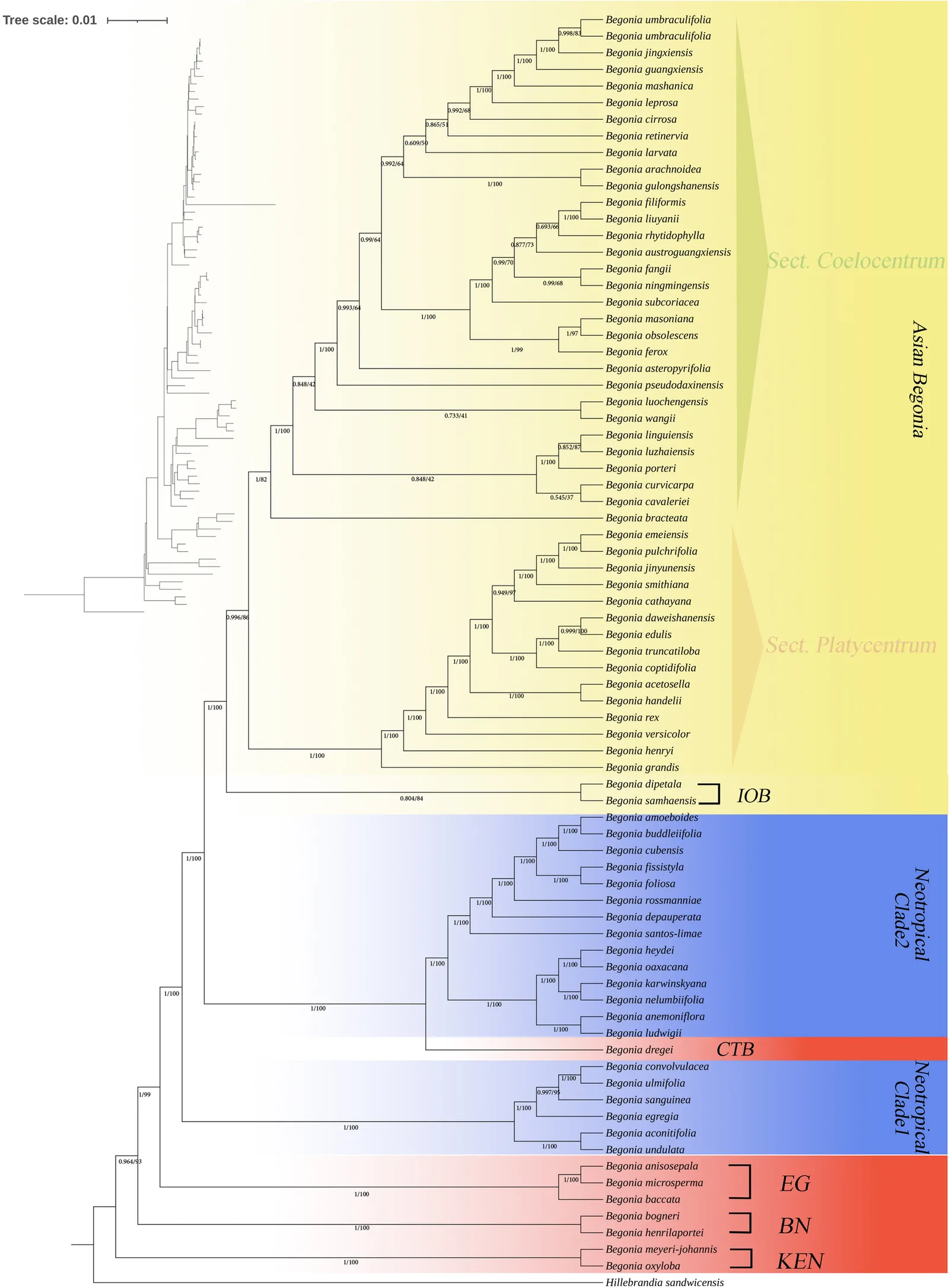
Phylogenetic tree inferred from the complete chloroplast genome sequences of 76 *Begonia* species using maximum likelihood (ML) and Bayesian inference (BI). Bootstrap support (ML, 1,000 replicates) and posterior probabilities (BI) are indicated at nodes (BS/PP). *Hillebrandia sandwicensis* was used as the outgroup. The numbers on the branches represent the posterior probabilities from BI and the ML bootstrap values

Phylogenetic analyses were performed on two independent datasets: (i) the 76 complete plastome sequences, and (ii) the 78 concatenated protein-coding genes (CDS) from the same species. Both datasets produced identical topologies with strong support for all major clades. Figure 7 shows the maximum likelihood tree inferred from the complete plastome dataset (Bayesian inference yielded an identical topology; tree not shown). The tree from the CDS dataset is provided in Fig. S2 for comparison. Most clades aligned with the geographic distributions of the species (Fig. 7, Fig. S2). African *Begonia* species (except *B. dregei*) formed three strongly supported clades: Kenyan *Begonia* (KEN: *B. oxyloba*, *B. meyeri-johannis*), Malagasy *Begonia* (MB: *B. henrilaportei*, *B. bogneri*), and Equatorial Guinea-region *Begonia* (EG: *B. anisosepala*, *B. baccata*, *B. microsperma*). These clades were positioned at the base to the phylogenetic tree (bootstrap support [BS] = 100, posterior probability [PP] = 1). American *Begonia* species clustered into two clades: Neotropical Clade 1 (NC1) and Neotropical Clade 2 (NC2). Notably, *B. dregei* (an African species) was positioned at the base of NC2 (BS = 100, PP = 1). Asian *Begonia* species included two sister clades: Sect. *Platycentrum* and Sect. *Coelocentrum* (BS = 100, PP = 1). Fifteen Sect. *Platycentrum* species formed a monophyletic group. Meanwhile, 28 species of Sect. *Coelocentrum*, three species of Sect. *Diploclinium* (*B. grandis*, *B. cavaleriei*, *B. mashanica*), and one species of Sect. *Bracteibegonia* (*B. bracteata*) clustered closely together—all of these species are distributed in southern or central China.

### Adaptive evolution of chloroplast genes

The vast majority (71/78) of chloroplast protein‑coding genes exhibited *Ka/Ks* ratios far below 1, indicating that purifying selection is the dominant evolutionary force. Likelihood ratio tests (LRTs) were performed for all genes with candidate BEB sites (Table S6). Only *ndhD* consistently showed *Ka/Ks* > 1 across all pairwise comparisons (maximum 2.541) and a significant LRT result (*P* = 0.0055). *matK* also showed a nominally significant LRT result (*P* = 0.022), although *Ka/Ks* exceeded 1 only in two species‑specific comparisons (1.0686). For *ndhB*, *rps8*, and *rps15*, LRTs were not statistically significant (*P* > 0.05), and their *Ka/Ks* > 1 values were sporadic (observed in only 3, 0, and 0 species pairs, respectively). Therefore, only *ndhD* shows consistent evidence for positive selection (*Ka/Ks* > 1 across all comparisons; LRT *P* = 0.0055). *matK* shows a nominally significant LRT (*P* = 0.022) but Ka/Ks > 1 in only two species pairs; *ndhB*,* rps8*, and *rps15* lack significant LRT support. We thus consider *ndhD* as the primary candidate for positive selection, while the other four genes are treated as candidates requiring further validation, with their sporadic signals possibly reflecting relaxation of purifying selection. These sporadic signals may reflect lineage-specific relaxation of purifying selection or stochastic variation rather than pervasive adaptive evolution across the genus.

**Fig. 8:**
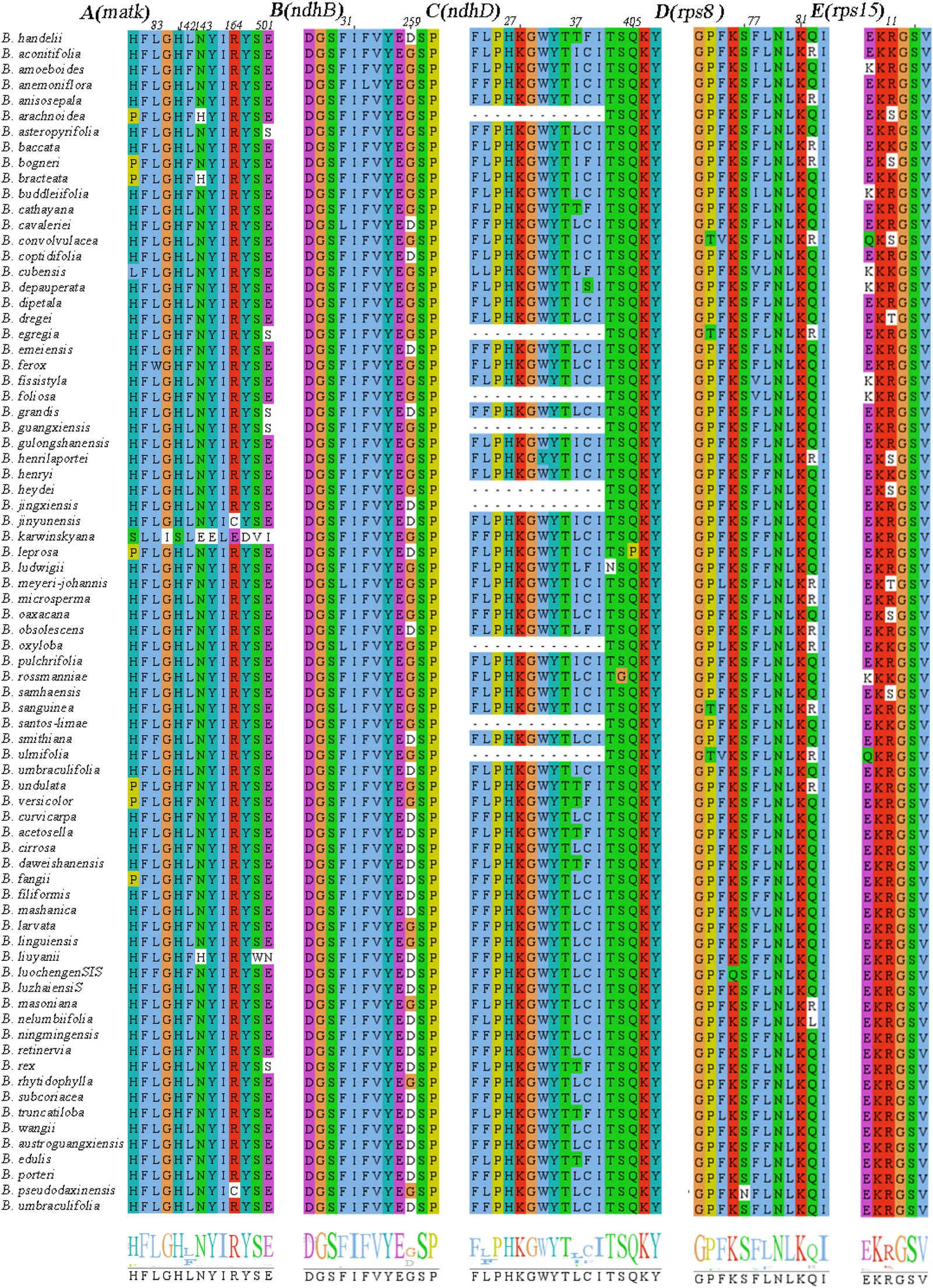
Comparison of partial sites under positive selection of different genes

**Fig. 9:**
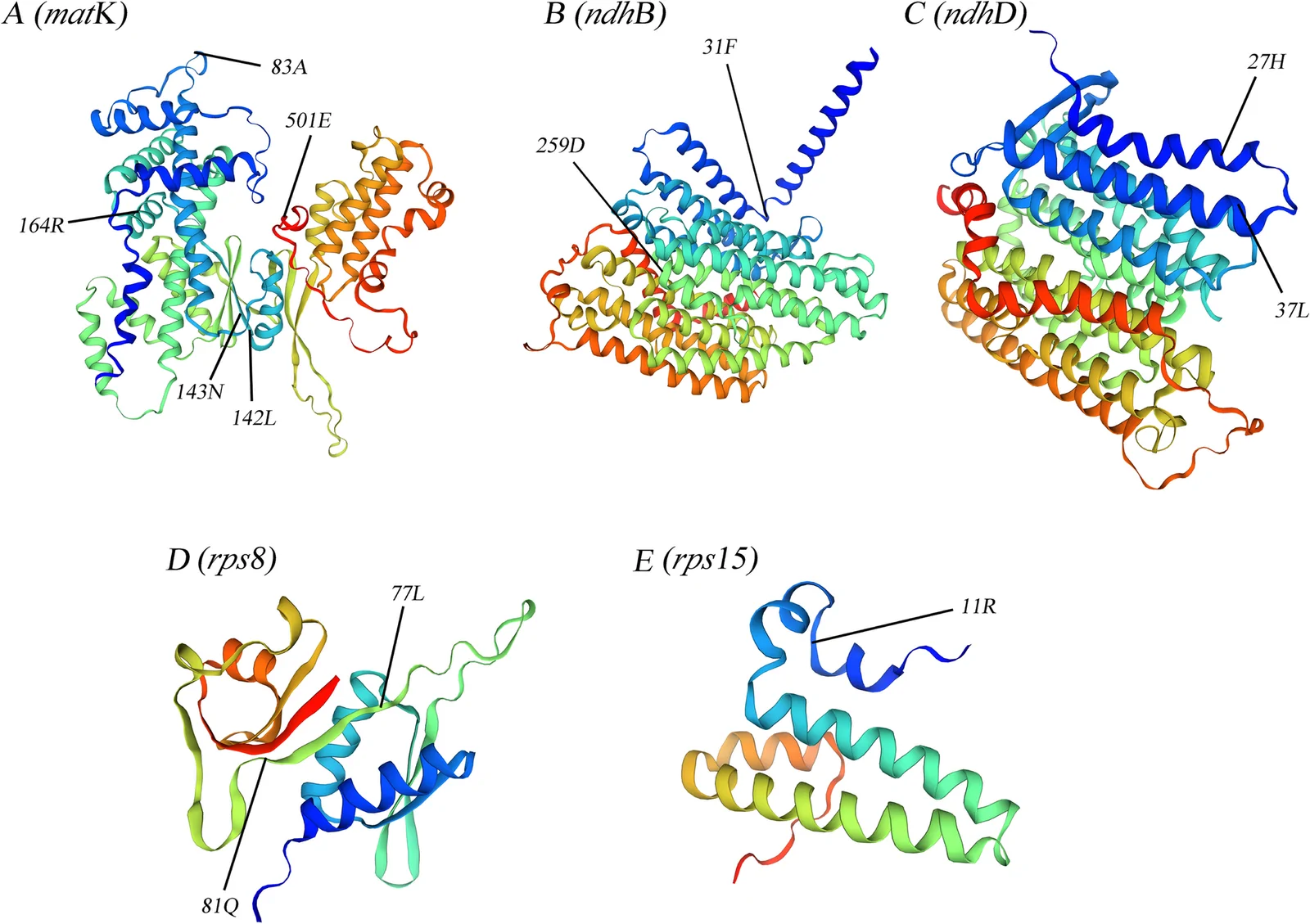
Spatial location of the positively selected sites in proteins

To further identify potential positively selected sites at the amino acid level, we applied the Bayes Empirical Bayes (BEB) test (posterior probability > 0.95) using EasyCodeML. This approach identified candidate sites in five genes (*matK*,* ndhB*,* ndhD*,* rps8*, and *rps15*). However, for *ndhB*,* rps8*, and *rps15*, the LRTs were not significant (Table S6), so these BEB-detected sites should be considered provisional and require independent validation. *matK*: 5 positively selected sites (83 A, 142 L, 143 N, 164R, 501E) located in random coils, β-sheets, and α-helices (Figs. 8A and 9A); *ndhB*: 2 positively selected sites (31 F in a random coil, 259D in an α-helix) (Figs. 8B and 9B); *ndhD*: 2 positively selected sites (27 H, 37I) within α-helices (Figs. 8C and 9C); *rps8*: 2 positively selected sites (77 L, 81Q) in random coils (Figs. 8D and 9D); *rps15*: 1 positively selected sites (11R) in a random coil (Figs. 8E and 9E). Amino acid sequences of these positively selected genes were visualized using Jalview (v10.0.7), and protein structures/domains were predicted via the SWISS-MODEL tool—with *B. acetosella* used as an example for structural visualization.

## Discussion

### Sequence variation in *Begonia* plastomes

Angiosperm plastomes typically span 120–160 kb [43, 44], however, the 25 *Begonia* plastomes analyzed here were larger (167,365 − 169,901 bp, average 169,145 bp). Overall GC content (35.42–35.89%) matches other angiosperms, yet IR regions show higher GC (38.33–38.98%), likely due to rRNA gene clustering [45], which enhances structural stability and explains why IRs are more conserved than SC regions. A notable lineage-specific trait of *Begonia* is the substantial expansion of its IR regions (~ 37.5 kb), much larger than the typical angiosperms IR (~ 25 kb) [46], indicating IR elongation is a unique evolutionary feature. Each *Begonia* plastome encodes 142–143 genes (92–93 CDSs, 42 tRNAs, 8 rRNAs), exceeding the gene count of most angiosperms [47, 48]. A distinctive trait is the location of *matK*: in most plants, *matK* lies in the LSC region [49], but in *Begonia* it resides within the *trnK-UUU* intron in the IR region, making it a multi-copy gene. This finding further confirms IR expansion and may explain the slower evolutionary rate of *matK* in *Begonia*.

### Insights from repeat sequence analysis

SSRs are widely used in population genetics due to their high polymorphism [50, 51]. We detected 148–191 SSRs per plastome across the 25 *Begonia* species, providing candidate markers for phylogeographic studies. Species-specific patterns were observed: pentanucleotide repeats occurred only in *B. masoniana* (AATAT/ATATT) and *B. acetosella* (AAAAT/ATTTT), highlighting lineage-specific microsatellite evolution. These divergent profiles suggest that chloroplast-derived SSR markers may require species-level validation before population-level application. SSRs have higher mutation rates than neutral DNA regions, making them critical for exploring genome evolution in *Begonia* [52, 53]. Repetitive sequences can protect protein-coding regions from mutagenic pressures by forming secondary structures or occupying genomic space [54]. Across the 25 plastomes, 1,880 repeats were found (Fig. 3), with forward and palindromic repeats most common. While 24 species contained all four repeat types (forward, palindromic, reverse, complementary), *B. pseudodaxinensis* uniquely retained only palindromic repeats. Interspecific differences in repeat number, type, and length provide a basis for developing lineage-specific markers to resolve taxonomic uncertainties and population-level divergences in *Begonia*.

### Patterns of codon usage bias

Codon usage bias affects gene expression, mRNA stability, and translational efficiency [55–57]. RSCU analysis across 25 *Begonia* plastomes revealed a strong and highly conserved bias toward A/U-ending codons (NNU/NNA) in all species, with avoidance of G/C-ending codons (NNG/NNC) (Fig. 5). This pattern is consistent with chloroplast genomes of other angiosperms [e.g., 47,48] and likely reflects two selective pressures: first, A/U-rich codons are preferentially recognized by plastid tRNAs, which are also A/U-rich in their anticodon regions, enhancing translation speed and accuracy [56]; second, A/U-rich coding sequences may increase mRNA stability in the chloroplast environment, where degradation is often initiated at GC-rich stem-loops [57].

The uniform codon preference across the 25 *Begonia* species—despite their diverse morphologies and ecological microhabitats—suggests that purifying selection, rather than adaptive divergence, dominates codon usage evolution in Begonia plastomes. This conservation indicates that efficient translation of chloroplast proteins is essential for basic photosynthetic function, and any deviation would impose a fitness cost.

The distinct codon usage profile of *B. pseudodaxinensis* (consistently lower RSCU values) is intriguing. This species also exhibited the lowest GC content in its LSC and SSC regions (Table 1) and uniquely lacked all complex repeats except palindromic ones (Fig. 3). These features may reflect a lineage-specific relaxation of selective constraints or a recent population bottleneck that allowed slightly deleterious mutations to drift to fixation. Alternatively, they could be artifacts of the single available accession; population-level sampling would be needed to distinguish between these hypotheses.

The observed lineage-specific variation in termination codon usage (UGA/UAA less frequent in five species) may affect translation termination efficiency, as UAA is the most efficiently recognized stop codon in plastids. Whether this variation has functional consequences or simply reflects neutral drift remains unknown.

### Identification of hypervariable region

Hypervariable regions with elevated sequence polymorphism are ideal for developing DNA barcodes. Universal barcodes like *matK* and *rbcL* vary in effectiveness across taxa [58–61], necessitating lineage-specific markers. Pi analysis identified four hypervariable genes in *Begonia* plastomes: *petB*, *rps16*,* ndhA*, and *ycf1*. Notably, *ycf1* has shown superior discriminatory power compared to *matK*,* rbcL*, and *trnH-psbA*, leading Dong et al. [62] to propose it as a core barcode for land plants. Our results position *ycf1* and *petB* as candidate markers for species delimitation and phylogeographic studies in *Begonia*. Seven intergenic spacers also exhibited high polymorphism: *atpF-atpH*,* rpoB-trnC-GCA*,* trnT-UGU-trnL-UAA*,* rbcL-accD*,* rps19-trnG-UCC*,* ycf1-ndhF*, and *ndhF-rpl32*. The latter two (located in the SSC region, Fig. 6) were previously recognized as hypervariable by Timme et al. [63], and Dong et al. [64] highlighted *ycf1-ndhF* and *rbcL-accD* for species-level phylogenetics. These spacers—especially those in the SSC region—are promising *Begonia*-specific DNA barcodes, combining high mutation rates with practical PCR feasibility.

### Resolving Phylogenetic Relationships in *Begonia*

With over 2,000 species, *Begonia* faces taxonomic challenges due to morphological convergence in flower, fruit, leaves, and rhizomes—traits subject to environmental plasticity. Plastomes (maternal inheritance, low recombination, and high conservation) have resolved complex phylogenies in Poaceae [65], Rutaceae [66], and *Camellia* [67]. Our phylogeny of 76 *Begonia* species using plastome and CDS data showed high topological consistency (Fig. 7, Fig. S2) and aligned with the geographic distributions, consistent with previous studies [68]. Our plastome‑based phylogeny differs from previous *Begonia* phylogenetic hypotheses in several topological features. Compared with the non‑coding plastid DNA phylogeny of Asian *Begonia* by Thomas et al. (2011) [12], our tree provides stronger support (BS = 100, PP = 1) for the monophyly of Sect. *Coelocentrum* and its sister relationship with Sect. *Platycentrum*, whereas Thomas et al. (2011) recovered a polyphyletic *Coelocentrum* with moderate support. Furthermore, the position of *B. mashanica* has been ambiguous in earlier studies based on morphology and limited molecular data [13]; our analysis unambiguously places *B. mashanica* within Sect. *Diploclinium* (BS = 100, PP = 1), sister to *B. grandis* and *B. cavaleriei*, a placement not explicitly recovered in previous plastid trees. Finally, the African species *B. drege*i was previously grouped with other African clades [13], but our tree places it at the base of Neotropical Clade 2 (BS = 100, PP = 1), suggesting a possible long‑distance dispersal event or incomplete lineage sorting that warrants further investigation. Sect. *Coelocentrum* species, predominantly distributed inGuangxi karst regions(dark, humid microhabitats like limestone rock walls, caves, or broadleaf forests), exhibit narrow endemism and fragmented distributions. Their plastomes remain highly conserved, likely due to similar growth environments.

### Adaptive Selection on chloroplast genes in *Begonia*

Most *Begonia* species are shade-tolerant, occupying low-light habitats (forests, valleys, and caves). Such conditions exert natural selection pressures that can increase mutation rates and leave selection signatures in chloroplast genes [69]; e.g. positive selection on *rpoC2* and *rbcL* in *Ardisia* species [70]. Using two approaches, we identified five genes under positive selection across 76 *Begonia* species: *matK*,* ndhB*,* ndhD*,* rps8*, and *rps15* (Figs. 8 and 9). *matK* encodes for an RNA splicing enzyme for processing type II introns in tRNA precursors (*trnK*,* trnA*,* trnI*, and *trnV*) and ribosomal protein genes (*rpl2* and *rps12-2*), essential for chloroplast function [13]; its adaptive evolution may reflect regulatory adjustments to environmental pressures. *ndhB* and *ndhD* code for NADH dehydrogenase subunits in the photosynthetic electron transport chain. However, these inferences must be tempered by the observation that 71 of 78 chloroplast genes are under strong purifying selection (*Ka/Ks* < < 1), and only *ndhD* consistently exceeded a *Ka/Ks* ratio of 1 across all comparisons. The sporadic *Ka/Ks* > 1 signals detected for *ndhB* and *matK* (in only 3 and 2 species pairs, respectively) are more parsimoniously explained by lineage‑specific relaxation of selective constraints or slightly deleterious mutations not yet removed by purifying selection, rather than by adaptive evolution. Therefore, we consider the five genes identified by BEB as candidates for further investigation, but definitive evidence of adaptation would require functional assays or population genomic scans. The concerted signals from these five genes may reflect a coordinated adaptive strategy in plastomes to cope with low-light conditions, optimizing photosynthesis-related functions while maintaining modular coordination. These findings provide new insights into chloroplast genome evolution and environmental adaptation in plants.

## Conclusions

This study conducted a systematic analysis of the plastomes from 25 *Begonia* species, yielding key insights into the genomic evolution and taxonomic utility of these organellar genomes within the genus. A primary finding was the high conservation of boundaries between IR and SC regions across the 25 plastomes—a pattern likely shaped by consistent evolutionary rates under shared environmental constraints. By examining codon usage patterns and adaptive selection signals, the research further clarified the evolutionary dynamics driving plastome divergence in *Begonia*. Phylogenetic trees constructed using plastomes effectively resolved interspecific relationships, confirming the utility of plastome for refining *Begonia* taxonomy. Additionally, the identified candidate SSR markers and hypervariable genomic regions represent valuable molecular resources for species identification and genetic diversity assessments, supporting investigations into evolutionary history and the mechanisms underlying its adaptation to diverse habitats, while also offering a molecular basis for germplasm conservation, species authentication, and genetic improvement of *Begonia*. Overall, this multi-dimensional analysis of genome structure, evolutionary traits, and hypervariable regions provides candidate genes for further functional studies on shade adaptation, while recognizing that purifying selection dominates plastome evolution in *Begonia*. Additionally, the analytical approaches employed here offer a useful reference framework for plastome studies in other medicinal plant groups, contributing to broader understanding of organellar genome evolution across angiosperms.

## Supplementary Information

Supplementary Material 1.

## Data Availability

The datasets generated and analyzed in this study are available in the GenBank of NCBI, and the complete chloroplast genome sequence of twenty-five Begonia species is deposited in GenBank of NCBI under accession number NC_088496, PQ619425-PQ619426, PQ675781-PQ675783, PQ827431-PQ827443, PQ827445, PV137934-PV137937, PV191313. The accession numbers for the remaining datasets used and analyzed in this study are listed in the Methods section and Supplementary Tables section. Supplementary data associated with this article can be found in the online version at doi:10.6084/m9.figshare.29899688.

## Abbreviations

Cp: Chloroplast
LSC: Large single copy
SSC: Small single copy
IR: Inverted repeat
PolyA: Polyadenine
PolyT: Polythymine
ML: Maximum likelihood
PI: Nucleotide diversity
Ka/Ks: Non-synonymous/synonymous substitution site
CDS: Cp-coding sequences
rRNA: Ribosomal RNA
tRNA: Transfer RNA
SSR: Simple sequence repeat
RSCU: Relative synonymous codon usage
