# Supplementary material for: Comparative genomics of Begonia chloroplasts: insights into molecular evolution, species identification, and phylogeny
Source: BMC Genomics. 2026 Jul 29;27:884. doi: 10.1186/s12864-026-13066-2 (PMC13700284; doi:10.1186/s12864-026-13066-2)
Supplement: Supplementary file 1 [file 12864_2026_13066_MOESM1_ESM.zip › Additional files/Figure S1.docx]

**Figure S1** 25 species of *Begonia.* A: *B. masoniana*; B*: B. retinervia*; C: *B. fangii*; D: *B. austroguangxiensis*; E: *B.porteri；*F: *B. subcoriacea*; G: *B. cirrose*; H: *B. liuyanii*; I: *B. luochengensis*; J: *B. ningmingensis*; K: *B. umbraculifolia*; L: *B. curvicarpa*; M: *B. wangii*; N: *B. filiformis*; O: *B. luzhaiensis*; P: *B. pseudodaxinensis*; Q: *B. rhytidophylla*; R: *B. linguiensis*; S: *B. larvata*; T: *B. edulis*; U: *B. rex*; V: *B. truncatiloba*; W: *B. daweishanensis*; X: *B. acetosella*; Y: *B. mashanica.*
