# Supplementary material for: Comparative genomics of Begonia chloroplasts: insights into molecular evolution, species identification, and phylogeny
Source: BMC Genomics. 2026 Jul 29;27:884. doi: 10.1186/s12864-026-13066-2 (PMC13700284; doi:10.1186/s12864-026-13066-2)
Supplement: Supplementary file 1 [file 12864_2026_13066_MOESM1_ESM.zip › Additional files/Figure S2.docx]

**Figure S2.** Phylogenetic tree of chloroplast genomes of *Begonia* species based on CDS datasets. Support values are shown for nodes as Bayesian inference posterior probability/maximum likelihood bootstrap.
