# Supplementary material for: Comparative genomics of Begonia chloroplasts: insights into molecular evolution, species identification, and phylogeny
Source: BMC Genomics. 2026 Jul 29;27:884. doi: 10.1186/s12864-026-13066-2 (PMC13700284; doi:10.1186/s12864-026-13066-2)
Supplement: Supplementary file 1 [file 12864_2026_13066_MOESM1_ESM.zip › Additional files/Table S1.docx]

**Table 1** Sources of material from 25 *Begonia* species.

| No. | Species | Sections | Introduction place | Voucher number | GenBank accession numbers |
| --- | --- | --- | --- | --- | --- |
| 1 | *B. retinervia* | *Coelocentrum* | Du 'an County, Hechi City, Guangxi | IBK-2024-110 | NC_088496 |
| 2 | *B. subcoriacea* | *Coelocentrum* | Daxin county, Chongzuo City, Guangxi | IBK-2024-151 | PQ619425 |
| 3 | *B. liuyanii* | *Coelocentrum* | Longzhou County, Chongzuo City, Guangxi | IBK-2024-152 | PQ619426 |
| 4 | *B. luochengensis* | *Coelocentrum* | Luocheng County, Hechi City, Guangxi | IBK-2024-153 | PQ675781 |
| 5 | *B. wangii* | *Coelocentrum* | Napo County, Bose City, Guangxi | IBK-2024-154 | PQ675782 |
| 6 | *B. filiformis* | *Coelocentrum* | Longan County, Nanning City, Guangxi | IBK-2024-155 | PQ675783 |
| 7 | *B. fangii* | *Coelocentrum* | Longzhou County, Chongzuo City, Guangxi | IBK-2024-156 | PQ827431 |
| 8 | *B. austroguangxiensis* | *Coelocentrum* | Longzhou County, Chongzuo City, Guangxi | IBK-2024-157 | PQ827432 |
| 9 | *B. cirrosa* | *Coelocentrum* | Napo County, Bose City, Guangxi | IBK-2024-158 | PQ827433 |
| 10 | *B. porteri* | *Coelocentrum* | Luocheng County, Hechi City, Guangxi | IBK-2024-159 | PQ827434 |
| 11 | *B. ningmingensis* | *Coelocentrum* | Du 'an County, Hechi City, Guangxi | IBK-2024-160 | PQ827435 |
| 12 | *B. umbraculifolia* | *Coelocentrum* | Longan County, Nanning City, Guangxi | IBK-2024-161 | PQ827436 |
| 13 | *B. curvicarpa* | *Coelocentrum* | Yongfu County, Guilin City, Guangxi | IBK-2024-162 | PQ827437 |
| 14 | *B. luzhaiensis* | *Coelocentrum* | Yangshuo County, Guilin City, Guangxi | IBK-2024-163 | PQ827438 |
| 15 | *B. edulis* | *Platycentrum* | Longzhou County, Chongzuo City, Guangxi | IBK-2024-164 | PQ827439 |
| 16 | *B. rex* | *Platycentrum* | Xishuangbanna in Yunnan province | IBK-2024-165 | PQ827440 |
| 17 | *B. truncatiloba* | *Platycentrum* | Estuary of Yunnan Province | IBK-2024-166 | PQ827441 |
| 18 | *B. daweishanensis* | *Platycentrum* | Dawei Mountain, Pingbian, Yunnan Province | IBK-2024-167 | PQ827442 |
| 19 | *B. acetosella* | *Platycentrum* | Xishuangbanna in Yunnan province | IBK-2024-168 | PQ827443 |
| 20 | *B. mashanica* | *Diploclinium* | Mashan county, Nanning City, Guangxi | IBK-2024-169 | PQ827445 |
| 21 | *B. larvata* | *Coelocentrum* | Jiangzhou District, Chongzuo City, Guangxi | IBK-2025-010 | PV137934 |
| 22 | *B. linguiensis* | *Coelocentrum* | Yongfu County, Guilin City, Guangxi | IBK-2025-011 | PV137935 |
| 23 | *B. masoniana* | *Coelocentrum* | Pingxiang City, Chongzuo City, Guangxi | IBK-2025-012 | PV137936 |
| 24 | *B. rhytidophylla* | *Coelocentrum* | Jingxi County, Bose City, Guangxi | IBK-2025-013 | PV137937 |
| 25 | *B. pseudodaxinensis* | *Coelocentrum* | Jingxi County, Bose City, Guangxi | IBK-2025-050 | PV191313 |
