# Supplementary material for: Comparative genomics of Begonia chloroplasts: insights into molecular evolution, species identification, and phylogeny
Source: BMC Genomics. 2026 Jul 29;27:884. doi: 10.1186/s12864-026-13066-2 (PMC13700284; doi:10.1186/s12864-026-13066-2)
Supplement: Supplementary file 1 [file 12864_2026_13066_MOESM1_ESM.zip › Additional files/Table S2.docx]

**Table S2** Summary of SSRs in 25 *Begonia* chloroplast genomes.

| Species | Mon | Di | Tri | Tetra | Penta | Hexa |
| --- | --- | --- | --- | --- | --- | --- |
| *B. masoniana* | 144 | 26 | 7 | 10 | 1 |  |
| *B. retinervia* | 143 | 23 | 5 | 10 |  |  |
| *B. fangii* | 149 | 24 | 6 | 10 |  |  |
| *B. austroguangxiensis* | 144 | 24 | 5 | 10 |  |  |
| *B. subcoriacea* | 148 | 24 | 5 | 11 |  |  |
| *B. cirrosa* | 144 | 23 | 3 | 11 |  | 1 |
| *B. liuyanii* | 146 | 24 | 5 | 11 |  |  |
| *B. luochengensis* | 140 | 24 | 5 | 12 |  |  |
| *B. porteri* | 144 | 23 | 5 | 11 |  |  |
| *B. ningmingensis* | 143 | 24 | 6 | 11 |  |  |
| *B. umbraculifolia* | 153 | 24 | 6 | 10 |  |  |
| *B. curvicarpa* | 154 | 22 | 2 | 11 |  |  |
| *B. wangii* | 154 | 22 | 6 | 9 |  |  |
| *B. filiformis* | 147 | 24 | 6 | 11 |  |  |
| *B. luzhaiensis* | 142 | 24 | 5 | 11 |  |  |
| *B. pseudodaxinensis* | 143 | 21 | 3 | 10 |  |  |
| *B. rhytidophylla* | 143 | 24 | 6 | 11 |  | 2 |
| *B. linguiensis* | 151 | 24 | 5 | 11 |  |  |
| *B. larvata* | 134 | 22 | 6 | 10 |  | 1 |
| *B. edulis* | 111 | 26 | 5 | 9 |  |  |
| *B. rex* | 128 | 26 | 3 | 11 |  | 1 |
| *B. truncatiloba* | 108 | 26 | 5 | 9 |  |  |
| *B. daweishanensis* | 111 | 26 | 5 | 9 |  |  |
| *B. acetosella* | 130 | 21 | 2 | 9 | 1 |  |
| *B. mashanica* | 149 | 22 | 6 | 9 |  |  |
