# Supplementary material for: Comparative genomics of Begonia chloroplasts: insights into molecular evolution, species identification, and phylogeny
Source: BMC Genomics. 2026 Jul 29;27:884. doi: 10.1186/s12864-026-13066-2 (PMC13700284; doi:10.1186/s12864-026-13066-2)
Supplement: Supplementary file 1 [file 12864_2026_13066_MOESM1_ESM.zip › Additional files/Table S4.docx]

**Table S4** Summary of complex repeats in chloroplast genomes of 25 *Begonia species*.

| Species | Palindrpmic（P） | Forward（F） | Complement（C） | Reverse（R） | Total |
| --- | --- | --- | --- | --- | --- |
| *B. masoniana* | 41 | 37 | 4 | 8 | 90 |
| *B. retinervia* | 31 | 31 | 1 | 5 | 68 |
| *B. fangii* | 33 | 34 | 4 | 7 | 78 |
| *B. austroguangxiensis* | 34 | 36 | 3 | 7 | 80 |
| *B. subcoriacea* | 34 | 32 | 4 | 9 | 79 |
| *B. cirrosa* | 33 | 33 | 3 | 3 | 72 |
| *B. liuyanii* | 31 | 32 | 1 | 2 | 66 |
| *B. luochengensis* | 28 | 29 | 3 | 6 | 66 |
| *B. porteri* | 35 | 35 | 3 | 6 | 79 |
| *B. ningmingensis* | 34 | 37 | 4 | 7 | 82 |
| *B. umbraculifolia* | 34 | 34 | 4 | 6 | 78 |
| *B. curvicarpa* | 40 | 38 | 6 | 4 | 88 |
| *B. wangii* | 33 | 32 | 1 | 3 | 69 |
| *B. filiformis* | 32 | 32 | 1 | 2 | 67 |
| *B. luzhaiensis* | 27 | 29 | 4 | 6 | 66 |
| *B. pseudodaxinensis* | 41 | 33 | 4 | 8 | 86 |
| *B. rhytidophylla* | 44 | 36 | 2 | 6 | 88 |
| *B. linguiensis* | 100 | 0 | 0 | 0 | 100 |
| *B. larvata* | 28 | 30 | 2 | 4 | 64 |
| *B. edulis* | 23 | 22 | 5 | 5 | 55 |
| *B. rex* | 32 | 32 | 2 | 2 | 68 |
| *B. truncatiloba* | 23 | 24 | 5 | 5 | 57 |
| *B. daweishanensis* | 23 | 22 | 5 | 5 | 55 |
| *B. acetosella* | 40 | 38 | 9 | 13 | 100 |
| *B. mashanica* | 36 | 36 | 3 | 4 | 79 |
